# Supplementary material for: Lysophosphatidic Acid Stimulates Mitogenic Activity and Signaling in Human Neuroblastoma Cells through a Crosstalk with Anaplastic Lymphoma Kinase
Source: Biomolecules. 2024 May 28;14(6):631. doi: 10.3390/biom14060631 (PMC11201523; doi:10.3390/biom14060631)

Journal : *Biomolecules*

Title: Lysophosphatidic acid stimulates mitogenic activity and signaling in human neuroblastoma cells through a crosstalk with anaplastic lymphoma kinase. **Authors**: Simona Dedoni, Maria C. Olianas, Pierluigi Onali

Supplementary table and figures

Table S1. Sequences of human ALK siRNA duplexes

SR300165A	
sense	**rGrCrArGrArArUrArCrArGrCrArCrCrCrArArArUrCrArAGA**
anti-sense	**rUrCrUrUrGrArUrUrUrGrGrGrUrGrCrUrGrUrArUrUrCrUrGrCrArG**
SR300165B	
sense	**rGrGrCrGrArGrCrUrArCrUrArUrArGrArArArGrGrGrArGGC**
anti-sense	**rGrCrCrUrCrCrCrUrUrUrCrUrArUrArGrURArGrCrUrCrGrCrCrCrU**
SR300165C	
sense	**rGrGrArArUrCrArCrCrArArCrArArArCrArUrGrCrCrUrUCT**
anti-sense	**rArGrArArGrGrCrArUrGrUrUrUrGrUrUrGrGrUrGrArUrUrCrCrArA**

where r = ribonucleotide. GA, GC and CT at the end of the sense sequences are deoxyribonucleotides.

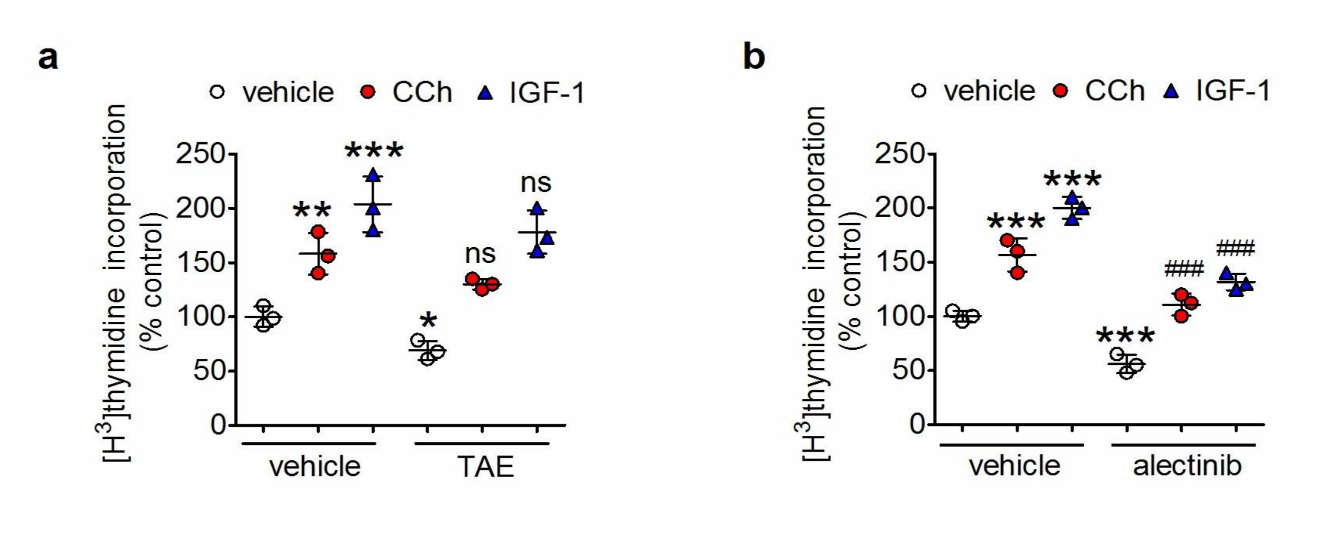

Figure S1. Effects of ALK inhibitors on stimulation of DNA synthesis by either CCh or IGF-1 in human neuroblastoma cells

Serum-starved SH-SY5Y cells were pretreated with either vehicle, 50 nM TAE684 (TAE) for 1 h (**a**), or 1 µM alectinib for 6 h (**b**), and then incubated for additional 24 h with either vehicle, 10 µM CCh or 20 ng/ml IGF-1 in the presence of [^{3}H]-thymidine. Values are expressed as percent of control (vehicle+vehicle) and the mean ± SD of three independent experiments. * $p < 0.05$, ** $p < 0.01$, *** $p < 0.001$ vs control; $^{###}$ $p < 0.001$ vs the corresponding sample without inhibitor; ns, not significantly different vs the corresponding sample without inhibitor by ANOVA followed by Neuman Keuls test.

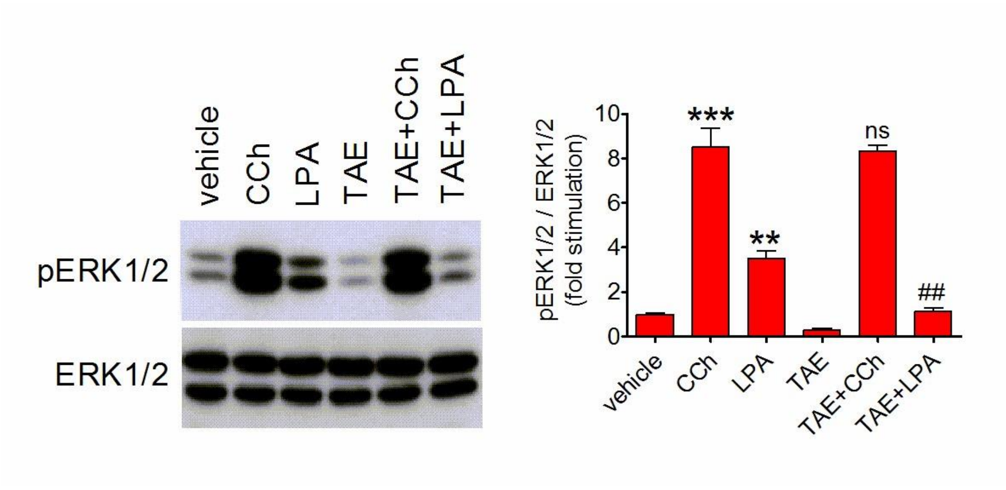

Figure S2. The ALK inhibitor TAE684 fails to affect ERK1/2 phosphorylation induced by CCh.

Serum-starved SH-SY5Y cells were pretreated for 1 h with either vehicle or 50 nM TAE684 (TAE) and then exposed for 5 min to either 10 µM CCh or 10 µM LPA. Cell lysates were analyzed for ERK1/2 phosphorylation by Western blot. Values are the mean ± SD of three independent experiments. ** $p < 0.01$, *** $p < 0.001$ vs control; ## $p < 0.01$ vs the LPA without inhibitor; ns, not significantly different from CCh without inhibitor by ANOVA followed by Neuman Keuls test.

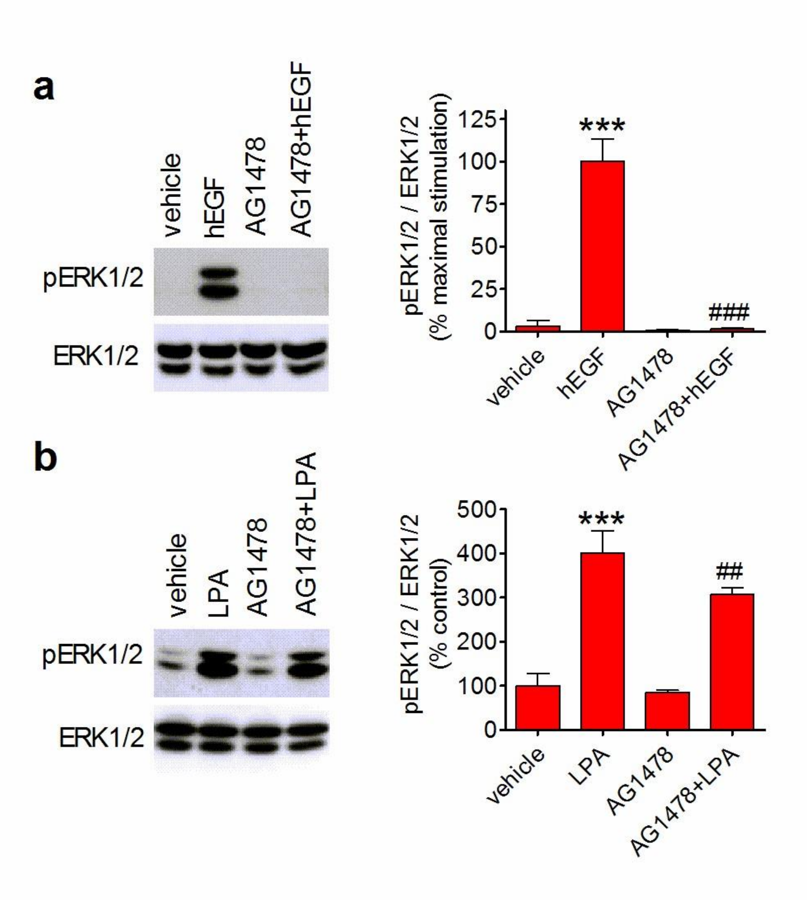

Figure S3. Effects of the EGF receptor tyrosine kinase inhibitor AG1478 on ERK1/2 activation by EGF and LPA in human neuroblastoma cells

Serum-starved SH-SY5Y cells were pretreated for 1 h with either vehicle or 200 nM AG1478 and then incubated for 5 min with either vehicle, 50 ng/ml human EGF (hEGF) (**a**) or 1 µM LPA (**b**), Cell lysates were analyzed for phospho-ERK1/2 (pERK1/2) and total ERK1/2 by Western blot. Values are the mean ± SD of three independent experiments. *** $p < 0.001$ vs control (vehicle+vehicle), $^{##}$ $p < 0.01$ vs vehicle + LPA, $^{###}$ $p < 0.001$ vs vehicle + hEGF by ANOVA followed by Neuman Keuls test.

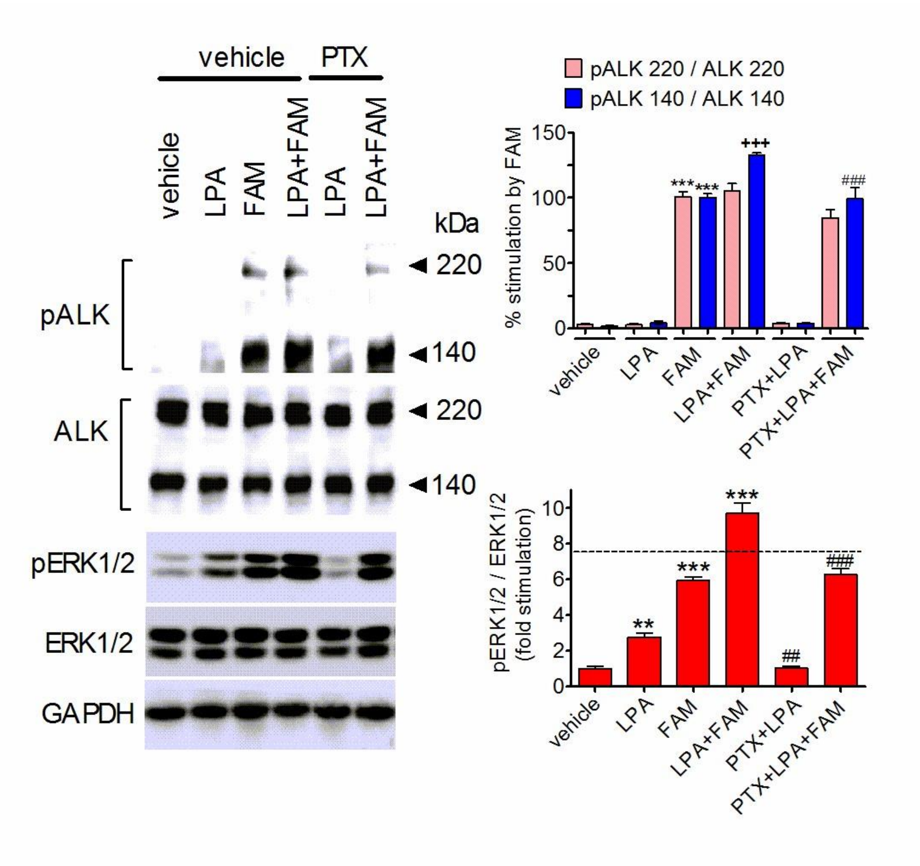

Figure S4. Involvement of PTX-sensitive G proteins in LPA-ALK crosstalk.

SH-SY5Y cells were incubated overnight in serum-free medium with either vehicle or PTX, pretreated for 5 min with either vehicle or 10 µM LPA and then incubated for 10 min with either control medium or FAM medium (FAM). Cell lysates were analyzed for phospho-ALK (Tyr1604) (pALK), total ALK, phospho-ERK1/2 (pERK1/2), total ERK1/2, and GAPDH. Values are the mean ± SD of three experiments. The dotted line indicates the level of pERK1/2 calculated by summing up the net stimulations by LPA and FAM medium alone. ** $p < 0.01$, *** $p < 0.001$ vs control (vehicle+vehicle), $^{\#\#}$ $p < 0.01$ vs LPA, $^{\#\#\#}$ $p < 0.001$ vs LPA+FAM, $^{+++}$ vs FAM by ANOVA followed by Neuman Keuls test.

Biomolecules – Supplementary information

Original images of Western blots

Figure 2

SH-SY5Y

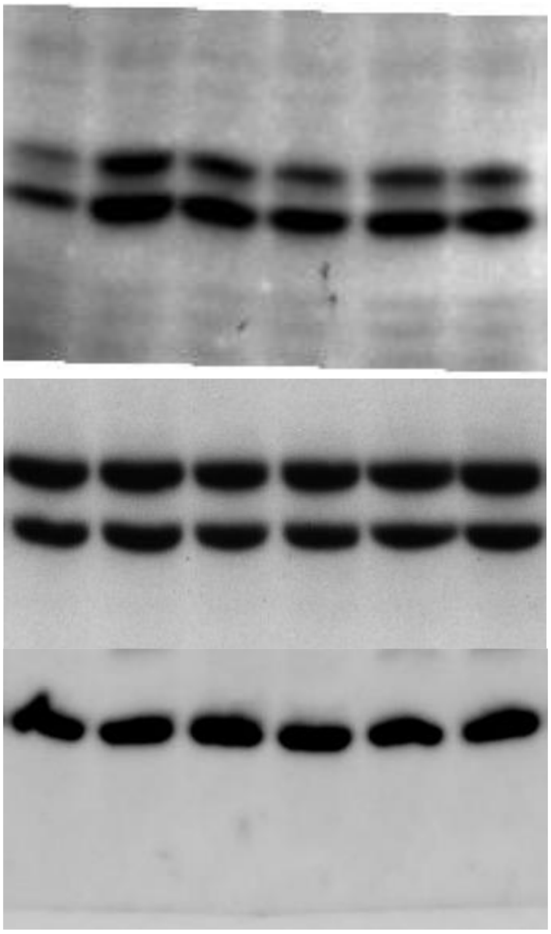

LAN-1

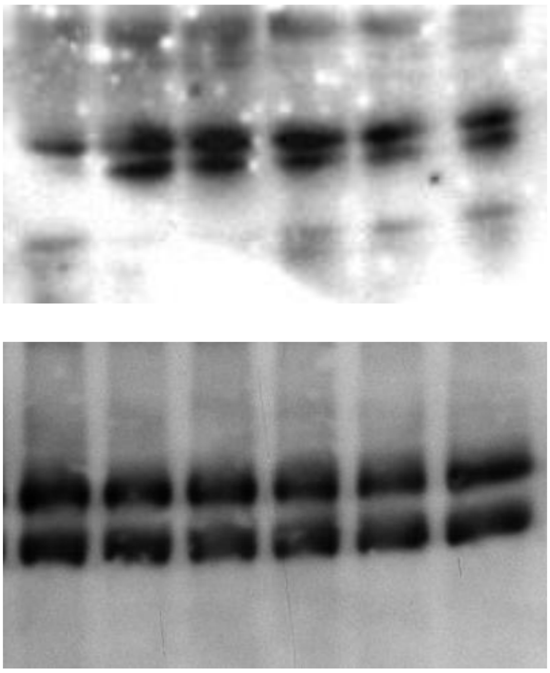

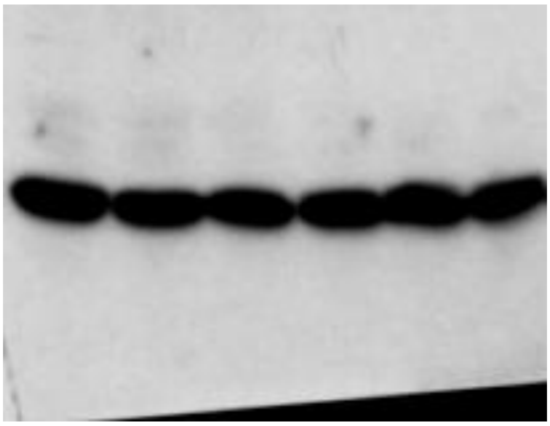

Kelly

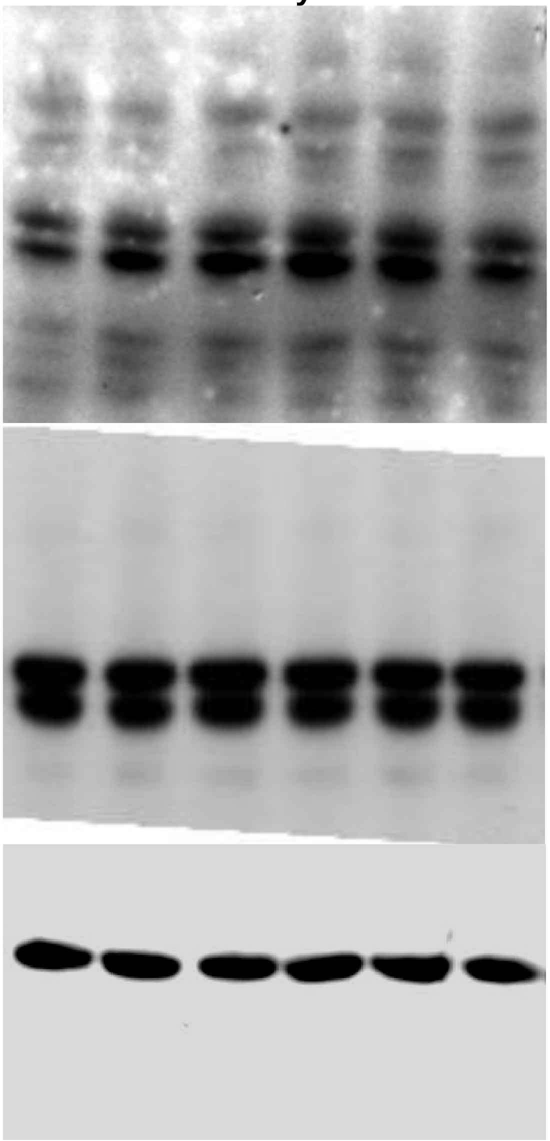

NB1

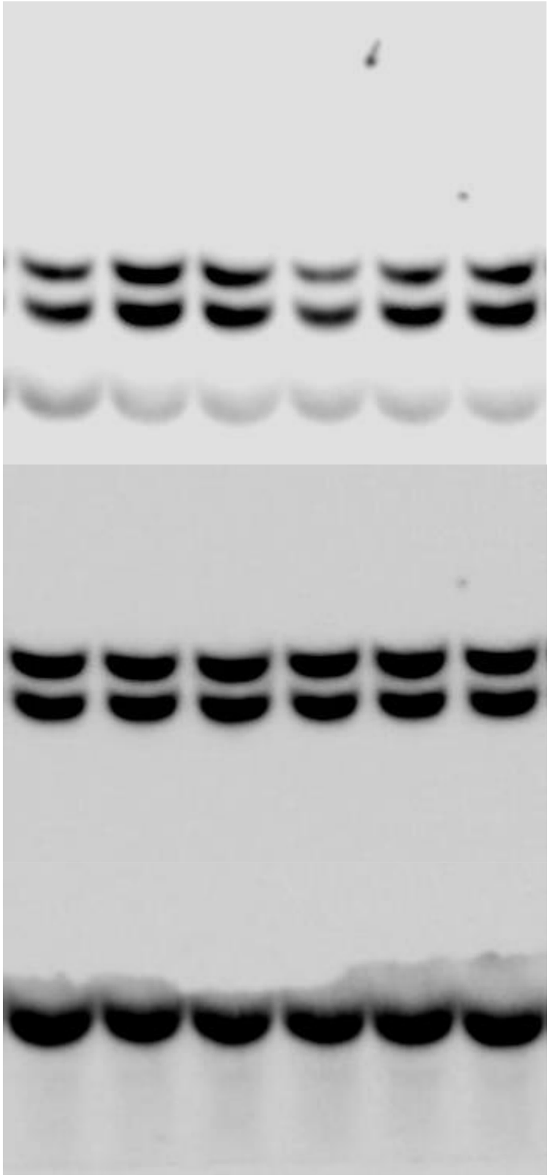

BE(2)C

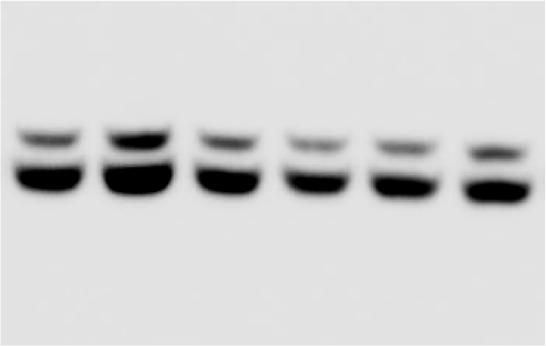

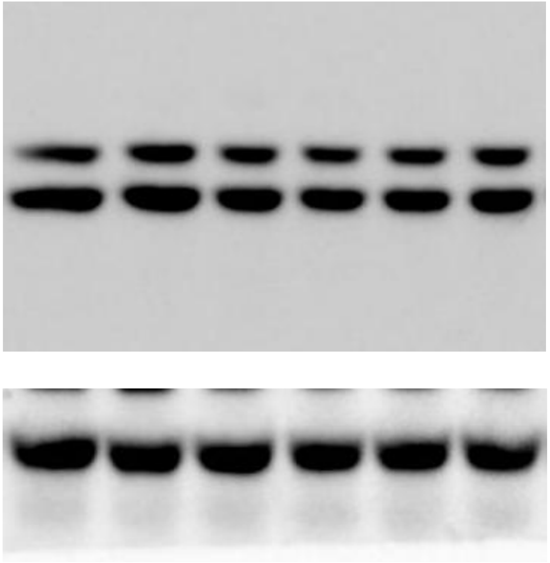

Figure 3a

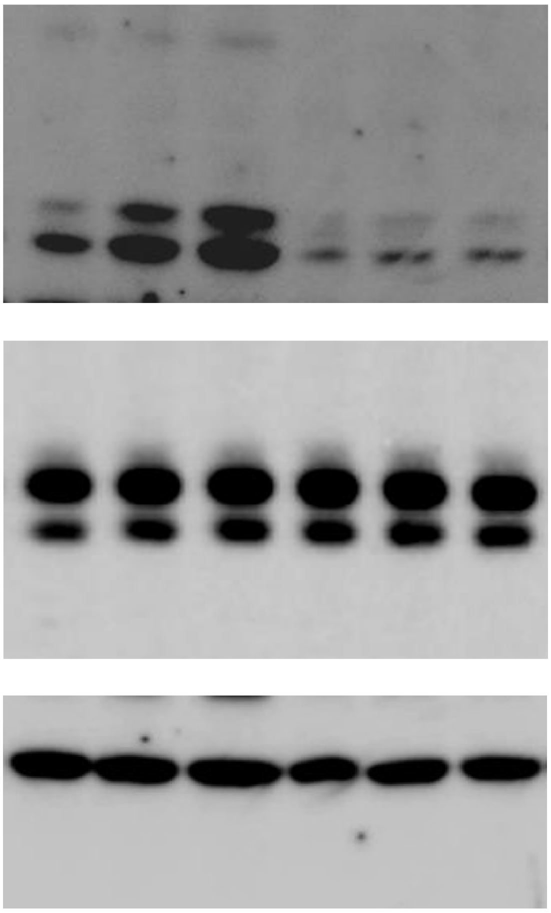

Figure 3b

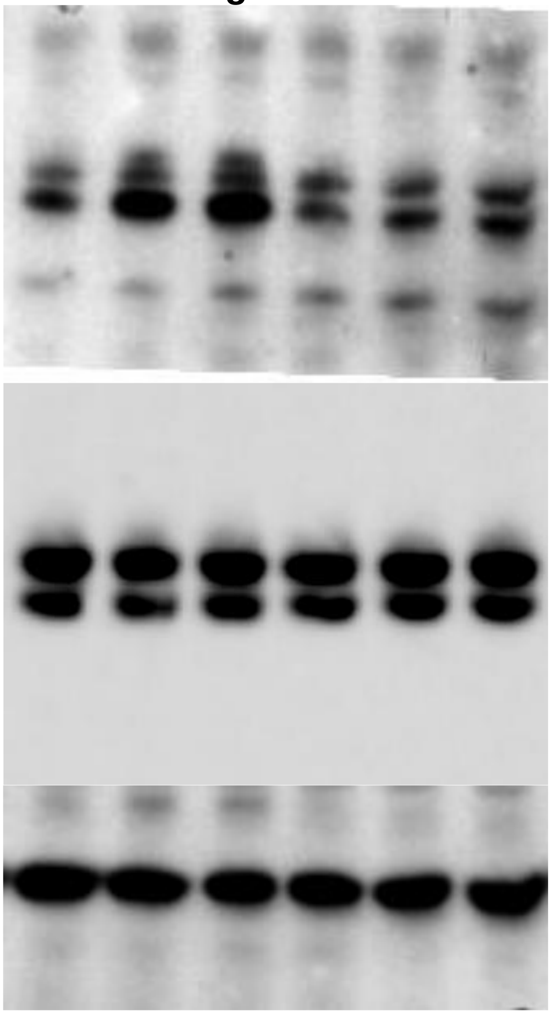

Figure 3c

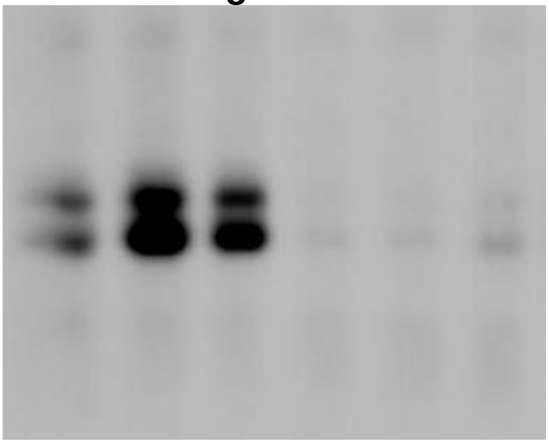

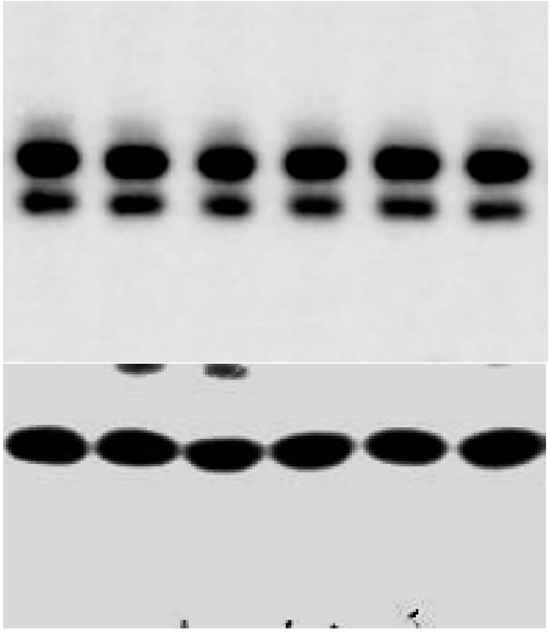

Figure 3d

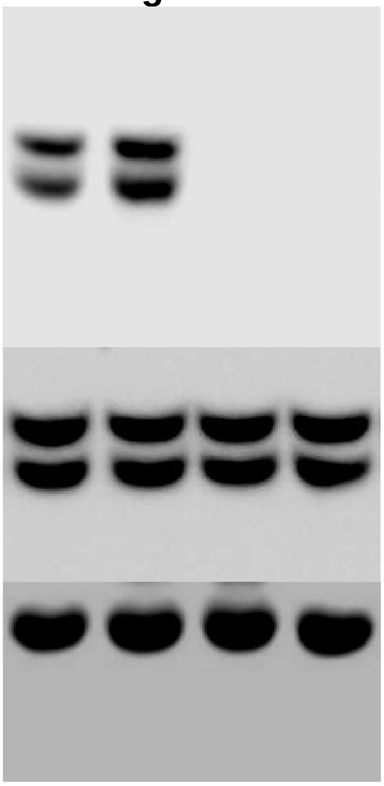

Figure 3e

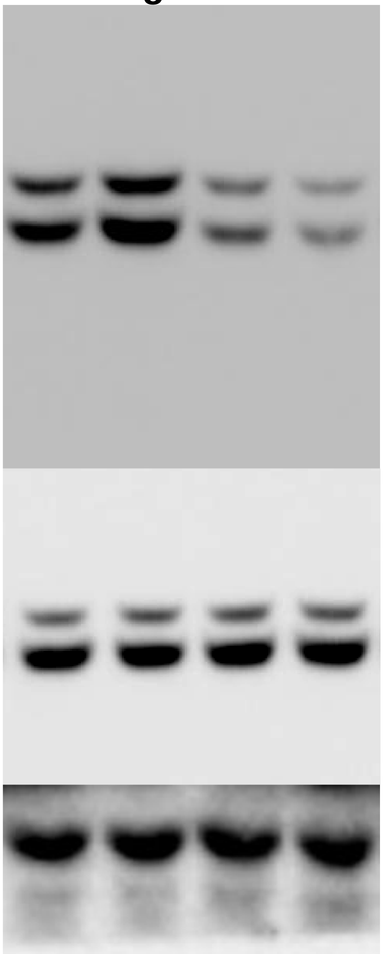

Figure 3f

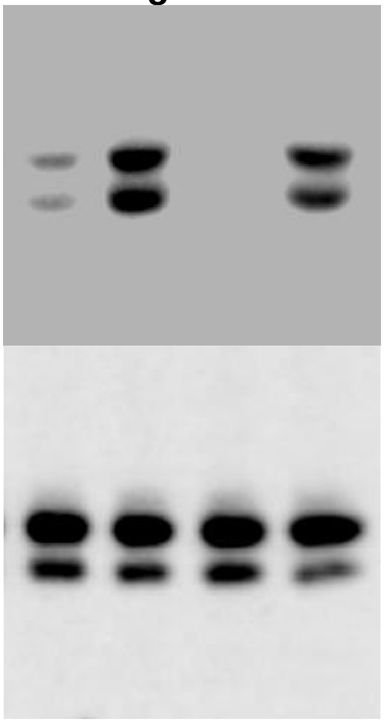

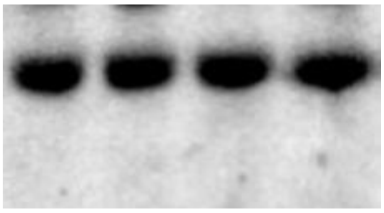

Figure 3g

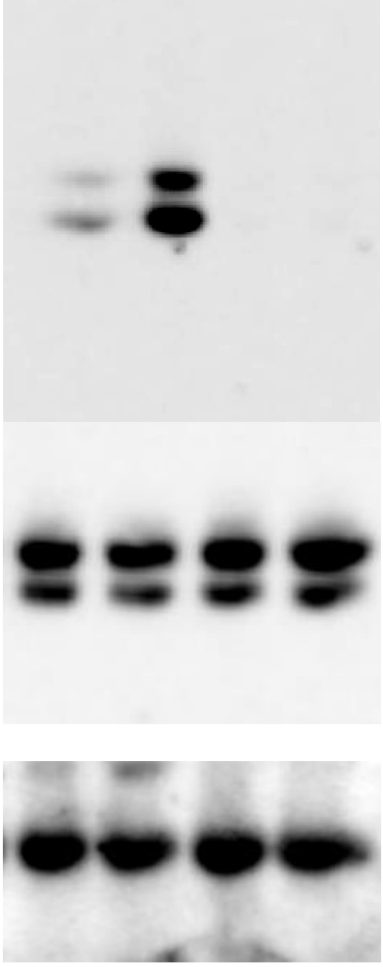

Figure 3h

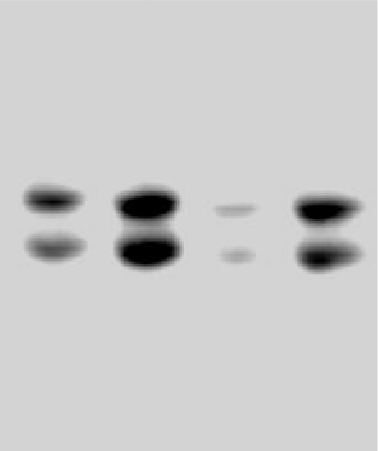

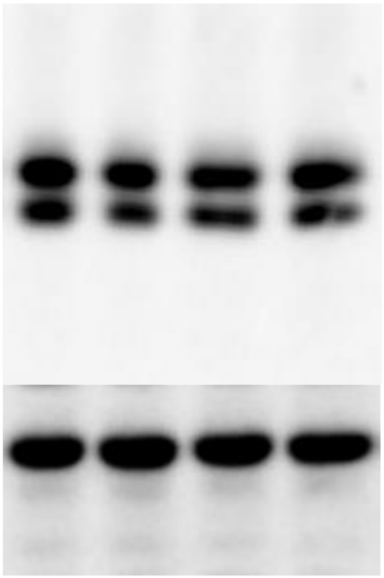

Figure 3i

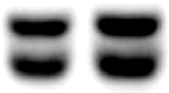

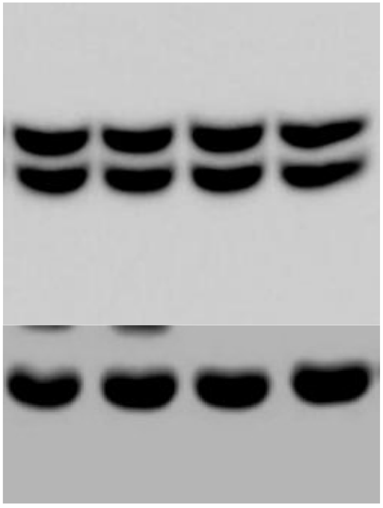

Figure 3j

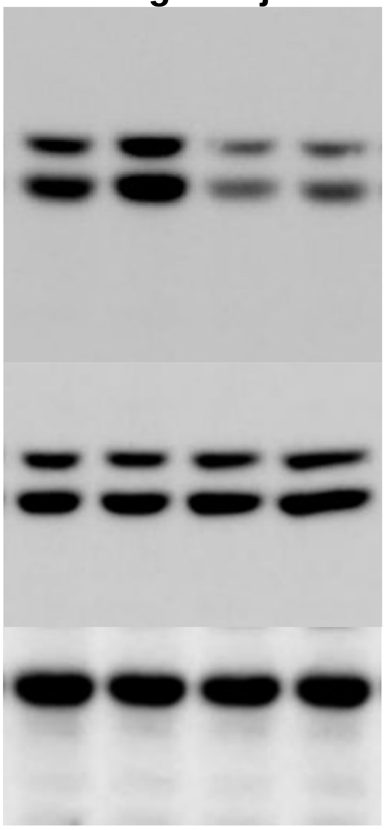

Figure 4a

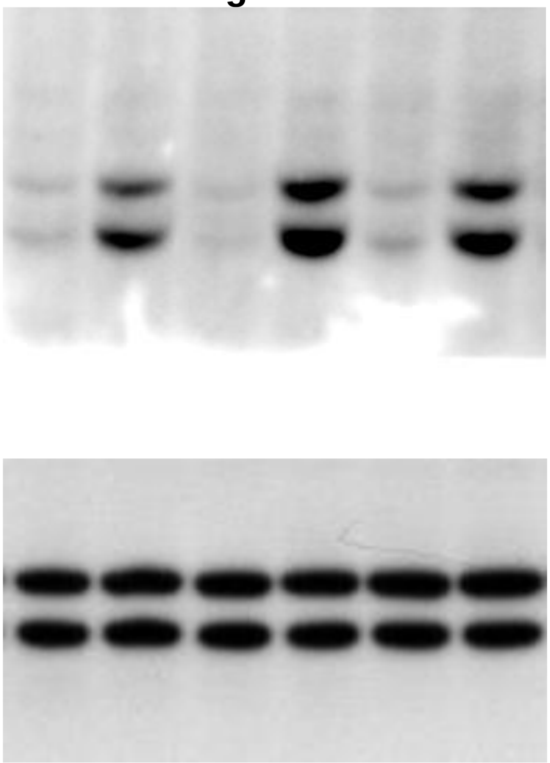

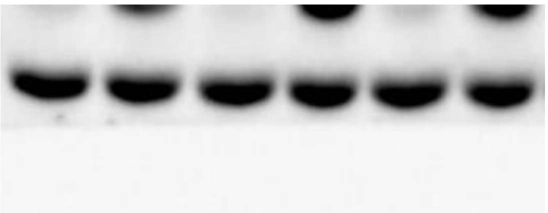

Figure 4b

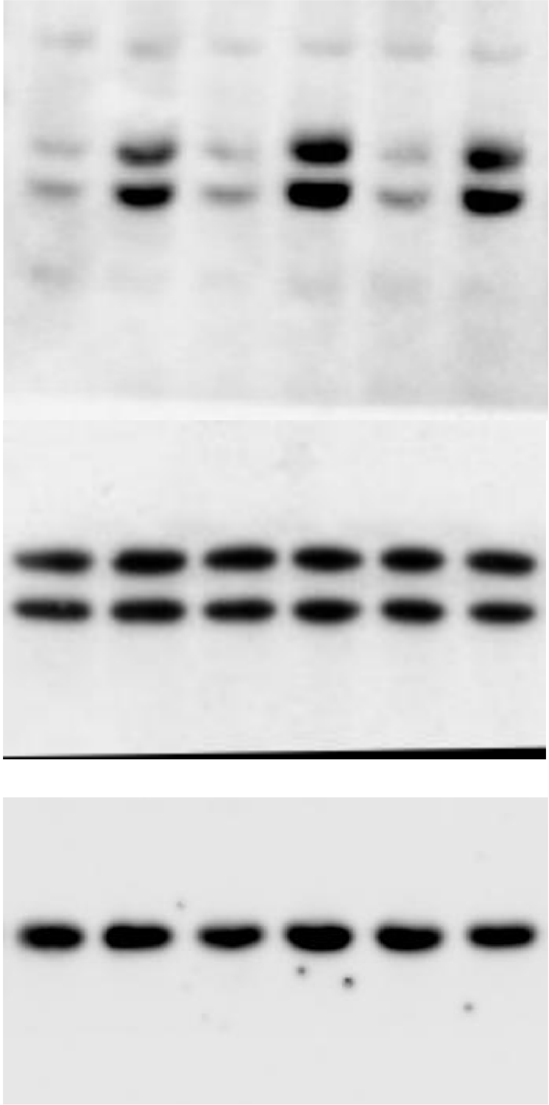

Figure 4c

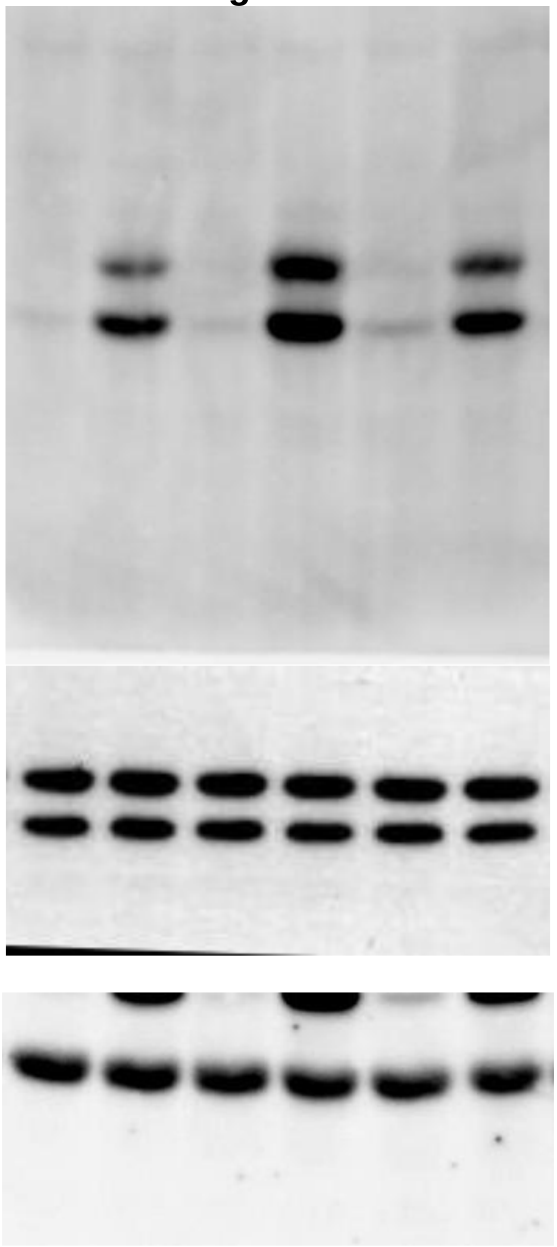

Figure 4d

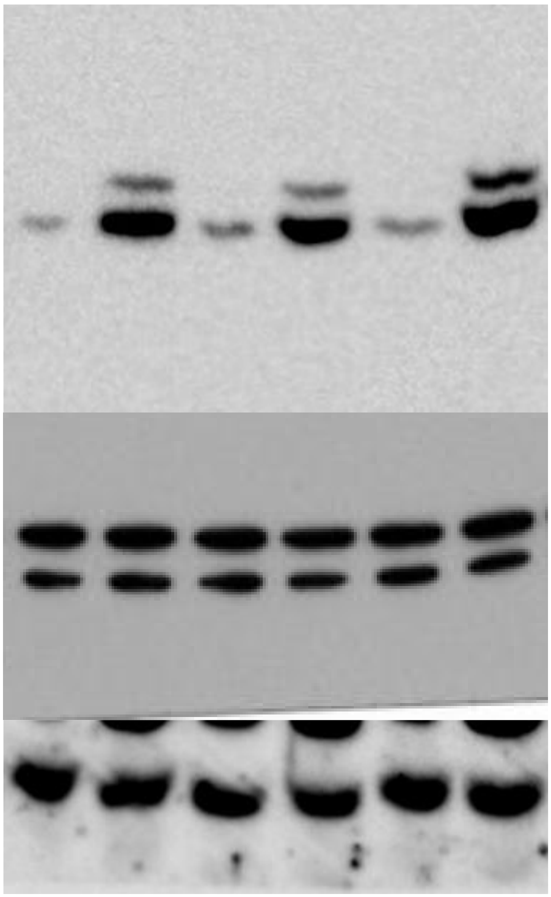

Figure 5a

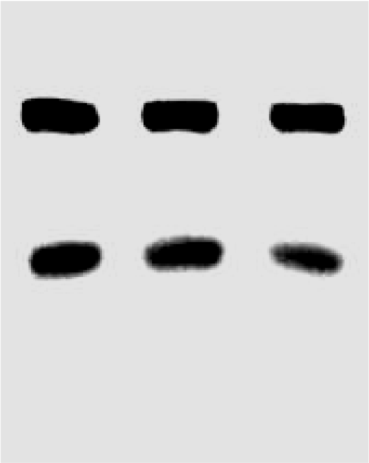

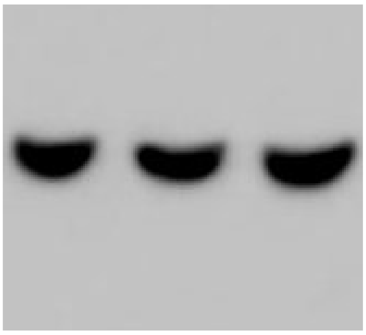

Figure 5b

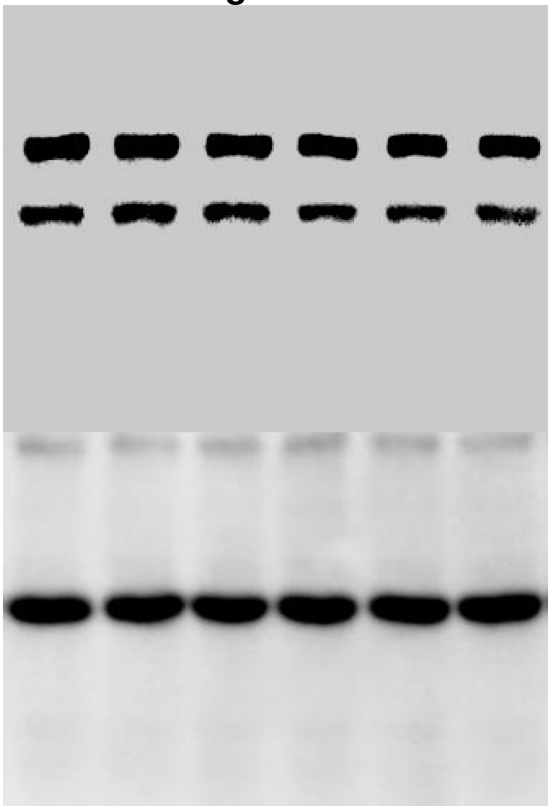

Figure 5c

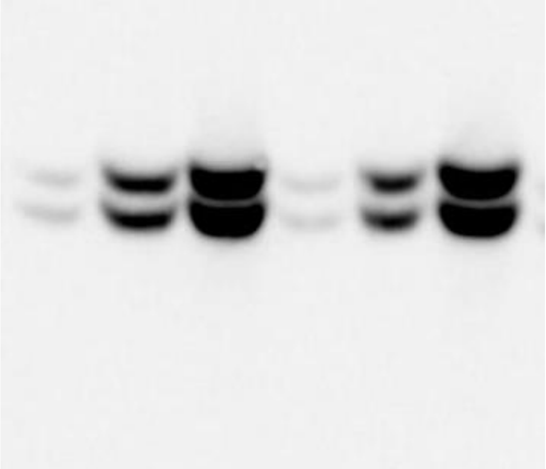

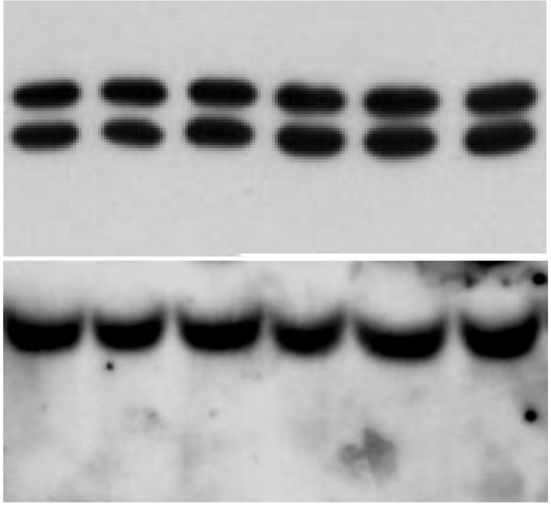

Figure 6a

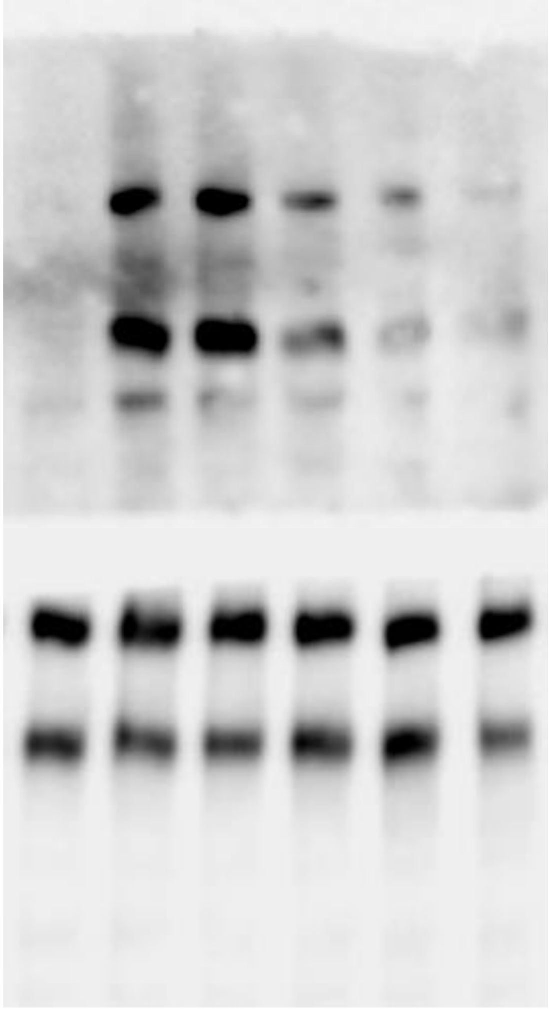

Figure 6b

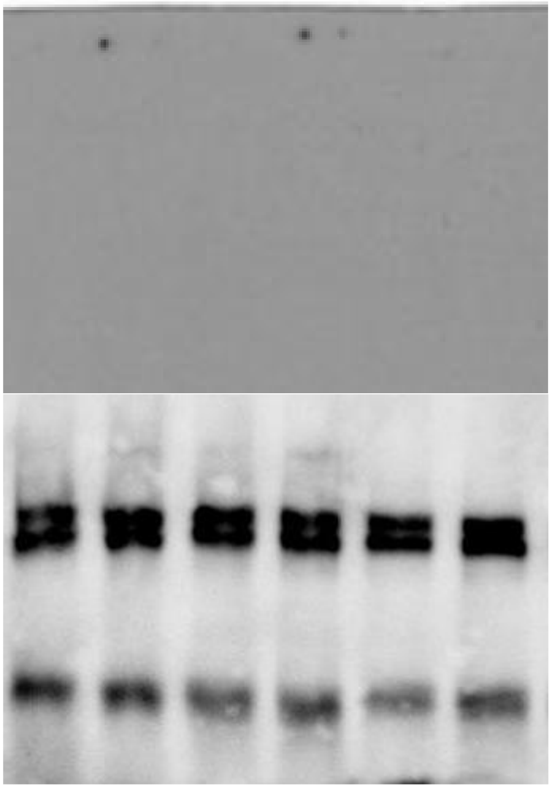

Figure 6c

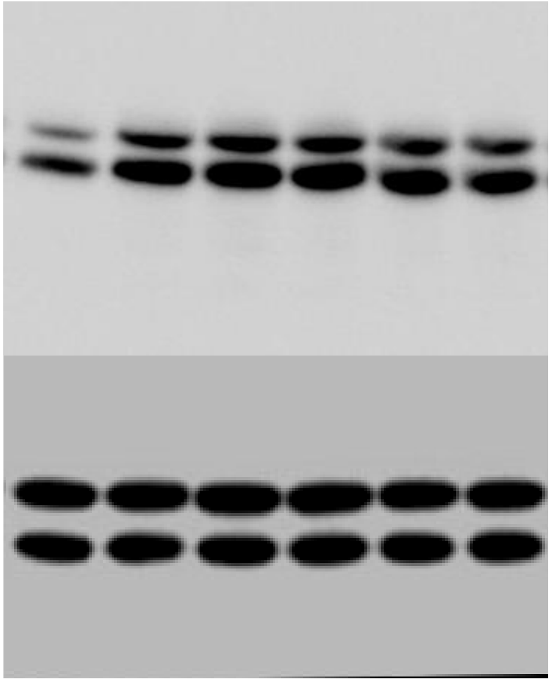

Figure 6d

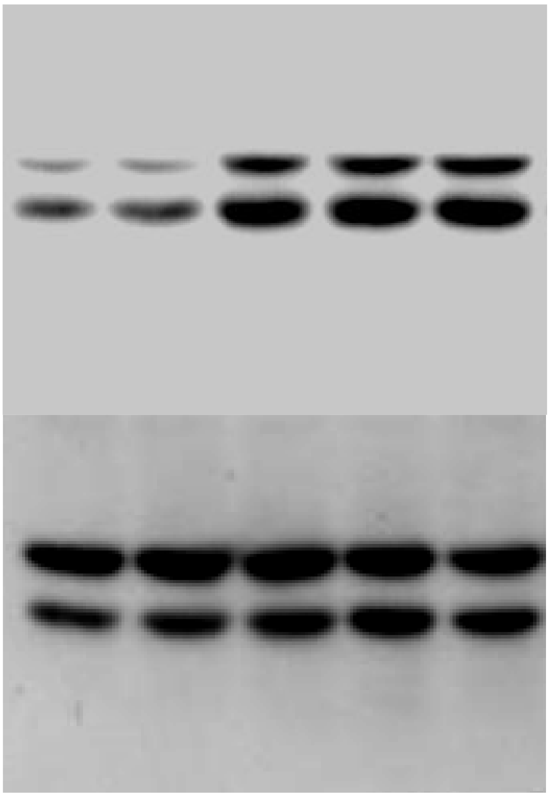

Figure 6e

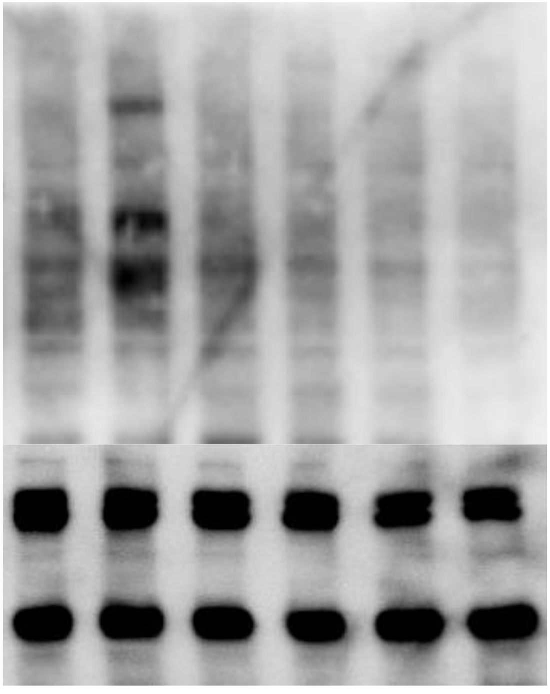

Figure 6f

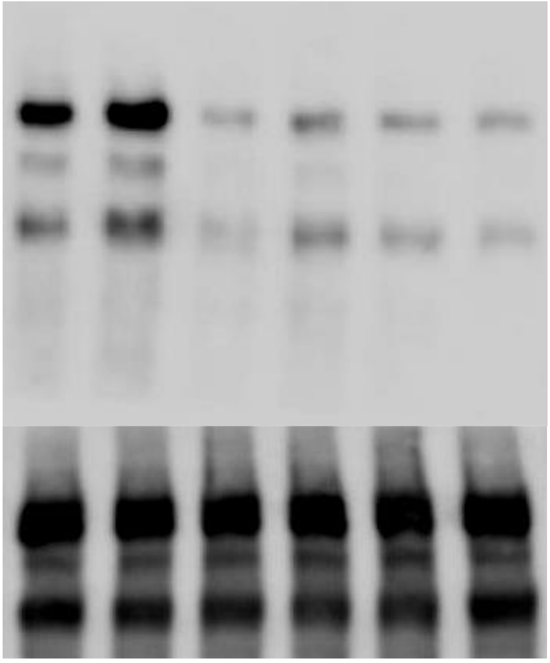

Figure 6g

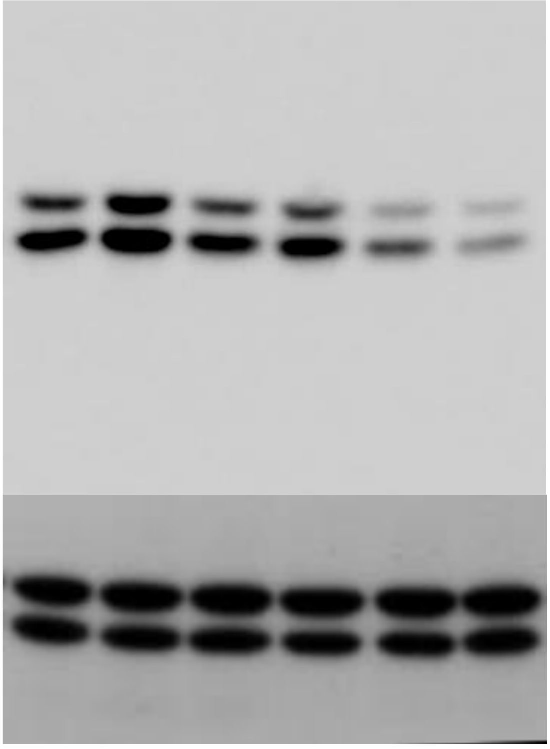

Figure 6h

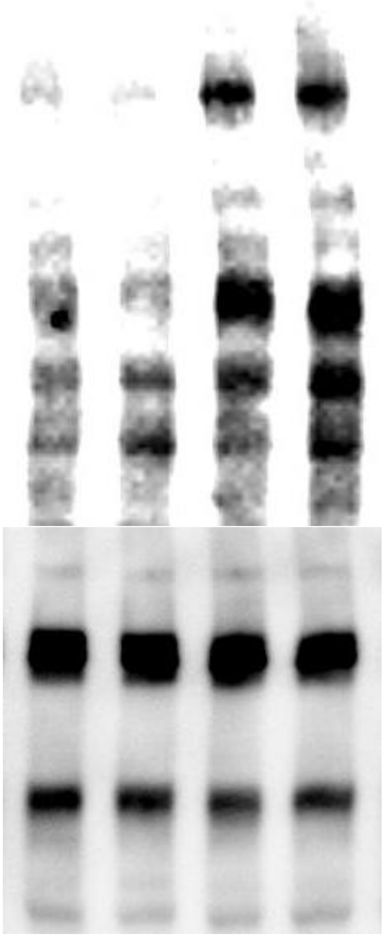

Figure 6i

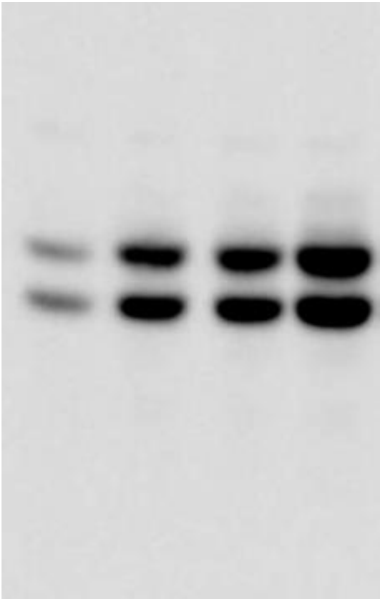

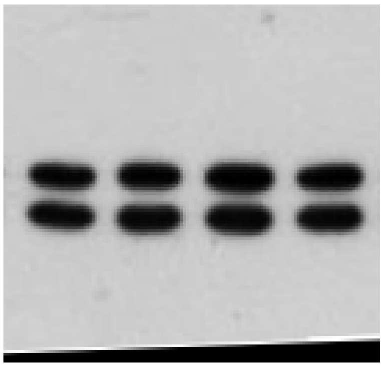

Figure 6j

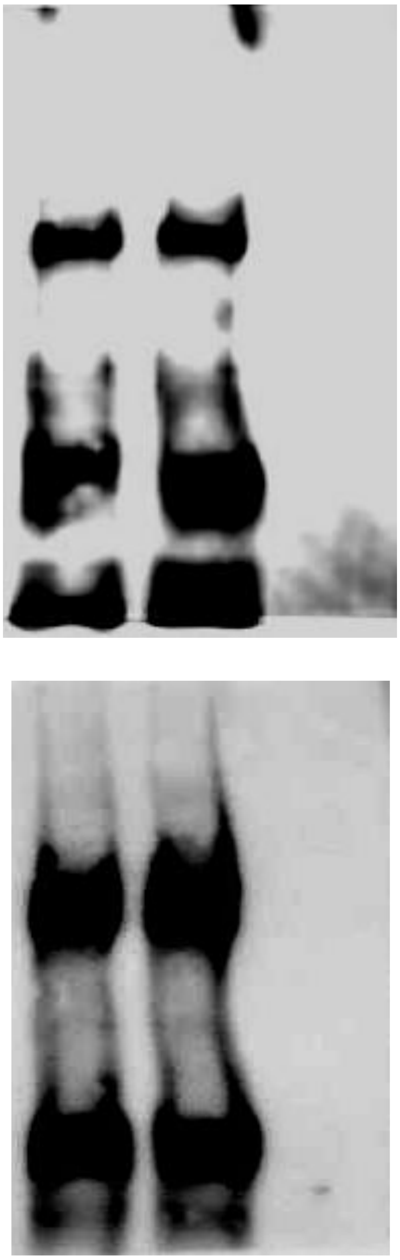

Figure 7a

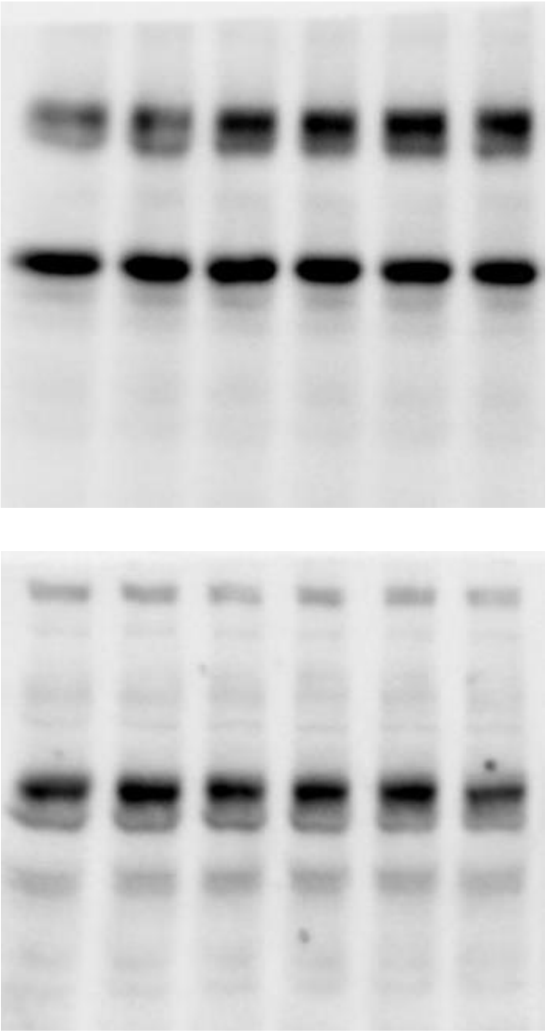

Figure 7b

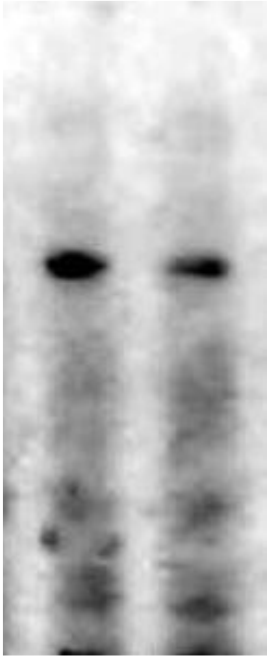

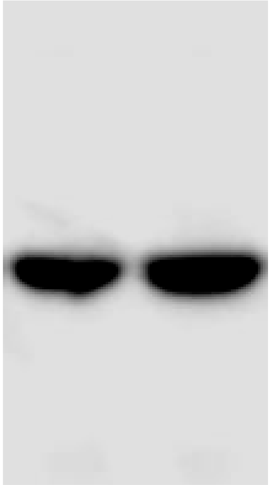

Figure 7c

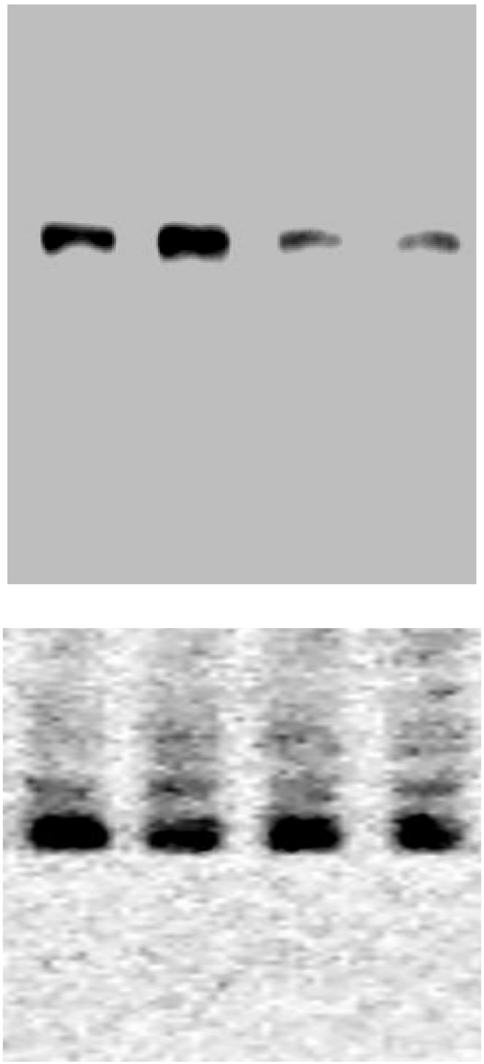

Figure 7d

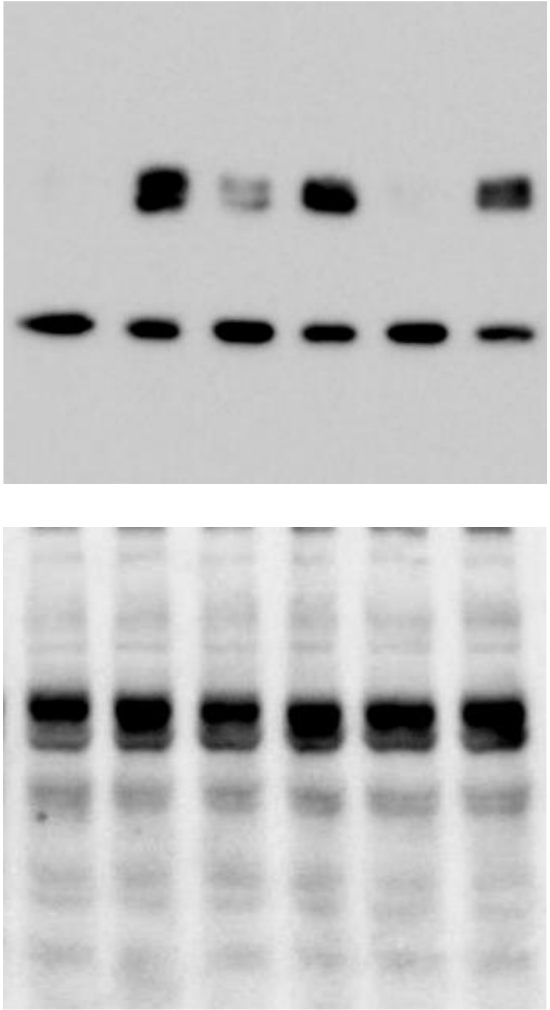

Supplement: Supplementary file 1 [file biomolecules-14-00631-s001.zip › biomolecules-2959460-supplementary.pdf]
